# Prevalence of Hepatitis B virus infection and its determinants among pregnant women in East Africa: Systematic review and Meta-analysis

**DOI:** 10.1371/journal.pone.0307102

**Published:** 2024-07-12

**Authors:** Bantie Getnet Yirsaw, Muluken Chanie Agimas, Gebrie Getu Alemu, Tigabu Kidie Tesfie, Nebiyu Mekonnen Derseh, Habtamu Wagnew Abuhay, Meron Asmamaw Alemayehu, Getaneh Awoke Yismaw

**Affiliations:** Department of Epidemiology and Biostatistics, Institute of Public Health, College of Medicine and Health Sciences, University of Gondar, Gondar, Ethiopia; Centre de Recherche en Cancerologie de Lyon, FRANCE

## Abstract

**Introduction:**

Hepatitis B virus (HBV) is one of the major public health problems globally and needs an urgent response. It is one of the most responsible causes of mortality among the five hepatitis viruses, and it affects almost every class of individuals. Different studies were conducted on the prevalence of HBV among pregnant women in East African countries, but none of them showed the pooled prevalence of HBV among the pregnant women. Thus, the main objective of this study was to determine the pooled prevalence and its determinants among pregnant women in East Africa.

**Methods:**

We searched studies using PubMed, Scopus, Embase, ScienceDirect, Google Scholar and grey literature that were published between January 01/2020 to January 30/2024. The studies were assessed using the Newcastle Ottawa Scale (NOS) quality assessment scale. The random-effect (DerSimonian) model was used to determine the pooled prevalence and associated factors of HBV among pregnant women. Heterogeneity were assessed by I^2^ statistic, sub-group analysis, and sensitivity analysis. Publication bias was assessed by Egger test, and the analysis was done using STATA version 17.

**Result:**

A total of 45 studies with 35639 pregnant women were included in this systematic review and meta-analysis. The overall pooled prevalence of HBV among pregnant women in East Africa was 6.0% (95% CI: 6.0%−7.0%, I^2^ = 89.7%). The highest prevalence of 8% ((95% CI: 6%, 10%), I^2^ = 91.08%) was seen in 2021, and the lowest prevalence 5% ((95% CI: 4%, 6%) I^2^ = 52.52%) was observed in 2022. A pooled meta-analysis showed that history of surgical procedure (OR = 2.14 (95% CI: 1.27, 3.61)), having multiple sexual partners (OR = 3.87 (95% CI: 2.52, 5.95), history of body tattooing (OR = 2.55 (95% CI: 1.62, 4.01)), history of tooth extraction (OR = 2.09 (95% CI: 1.29, 3.39)), abortion history(OR = 2.20(95% CI: 1.38, 3.50)), history of sharing sharp material (OR = 1.88 (95% CI: 1.07, 3.31)), blood transfusion (OR = 2.41 (95% CI: 1.62, 3.57)), family history of HBV (OR = 4.87 (95% CI: 2.95, 8.05)) and history needle injury (OR = 2.62 (95% CI: 1.20, 5.72)) were significant risk factors associated with HBV infection among pregnant women.

**Conclusions:**

The pooled prevalence of HBV infection among pregnant women in East Africa was an intermediate level and different across countries ranging from 1.5% to 22.2%. The result of this pooled prevalence was an indication of the need for screening, prevention, and control of HBV infection among pregnant women in the region. Therefore, early identification of risk factors, awareness creation on the mode of transmission HBV and implementation of preventive measures are essential in reducing the burden of HBV infection among pregnant women.

## Introduction

Globally, hepatitis B virus (HBV) is one of the major public health problems that can cause both acute and chronic diseases [1–3], and that requires an urgent response.

Among the five viruses (hepatitis A, B, C, D, and E virus), which are responsible for most cases of viral hepatitis, HBV and hepatitis C virus cause 96% of the mortality from these viral hepatitis. In 2015, around 257 million people were living with the Hepatitis B virus, and among these, 68% of the infected persons were from Africa and Western Pacific regions [4]. Specifically, the burden of the HBV is still high in eastern African countries, and it needs the implementation of universal and free HBV vaccination for all adults in the region [5].

Sero-positivity with HBV in pregnant women can transmit the disease to an unborn child during pregnancy. From a total of the world’s population, 25.3% are women reproductive age. Of those chronically infected, there are 65 million women who are of childbearing age who can potentially transmit HBV to their babies [4].

Results showed that, the prevalence of HBV among pregnant women is still high according to the WHO classification [1, 6]. The pooled prevalence of HBV among pregnant women in Ethiopia 4.7% [7] and 4.75% [8] which is an intermediate level of HBV, but individual studies showed that the prevalence is still beyond these pooled values[9–11].

So many studies have been conducted on the prevalence of the HBV among pregnant women in each East African country separately. For each separate study different key risk factors were identified for pregnant women, like abortion history [12–23]), History of multiple sexual partners ([1, 15, 18, 19, 21–30], surgical procedure [14, 24, 26, 31], History of hospitalization [2, 10, 12, 15, 16, 19, 20, 31, 32], traditional tonsillectomy [1, 2, 19], history of sexually transmitted infection [2, 30], human immunodeficiency virus (HIV) [2, 10, 16, 29, 33], alcohol drinking [2, 15], history of blood transfusion [14, 16–18, 21, 29, 34–37], knowledge about HBV [34], female genital mutilation [24, 34], frequency of testing & screening [34], tattooing[1, 10, 13, 21, 25, 28, 29, 31, 35], tooth extraction/dental therapy [14, 15, 24, 28, 31, 35], History of home delivery, insufficient knowledge and working at health facility [10], sharing sharp materials [10, 25, 26], history of vulvar ulcer [36], history of contact with patients who had jaundice [1, 36], history of contact with liver disease person[15], the age of the pregnant women [16, 29], education [17, 29], family history of HBV [17, 25, 26, 29, 32], Parity [17, 33], home delivery by traditional birth attendants [18] were significant risk factors for HBV infection among pregnant women. But on the other side, different studies showed risk factors such as: multiple sexual partners [38–41], HIV serostatus [38], history of blood transfusion [38, 39, 41], surgical procedure [39, 41], hospital admission, genital mutilation, body tattooing, and history of previous birth delivery [39], history of abortion and History of sharing sharp materials [40], history of dental procedure [41] were not statistically significant risk factors for HBV infection among pregnant women. Therefore, results showed inconsistent outputs on the risk factors for HBV among pregnant women, and therefore, this study figured out the key risk factors for HBV among pregnant women using systematic and meta-analyses in East Africa. This could contribute a significant role to achieve the sustainable development goal 3, which focuses on fighting communicable diseases such as HBV to eliminate viral hepatitis by 2030[4].

But to the best of the researchers knowledge, there was no study conducted using systematic review and meta-analysis for the prevalence of the HBV and its determinants among pregnant women in East Africa.

Thus, the objective of this study was to determine the pooled prevalence and its determinants among pregnant women in this area.

## Methods

### Study protocol and registration

This study was conducted on the prevalence of HBV and its determinants among pregnant women in East Africa, and conducted following guidelines of the Preferred Reporting Items for Systematic Reviews and Meta-Analysis (PRISMA). The study has been registered on the International Prospective Register of Systematic Review (PROSPERO), with registration number CRD42024512759.

### Searching strategy

We searched studies that were published between January 01/2020 to January 30/2024 using electronic databases such as PubMed, Scopus, Embase, ScienceDirect, Google Scholar, and other sources. Key words like Hepatitis B, prevalence, determinants and East Africa with their corresponding Medical Subject Headings (MeSH) terms were used to search by combining using Boolean operators (AND, OR, NOT). For instance, the advanced PubMed search strategy was: (((Hepatitis B OR Hepatitis B virus OR HBV OR Hepatitis OR Hepatitis B Virus Infection) AND (Prevalence OR Magnitude OR Proportion OR Burden OR Epidemiology)) AND (Determinants OR Predictor OR Risk Factor OR Associated Factor OR Related Factor)) AND (East Africa OR Eastern Africa OR Eritrea OR Ethiopia OR Djibouti OR South Sudan OR Rwanda OR Uganda OR Burundi OR Malawi OR Zimbabwe OR Zambia OR Mozambique OR Madagascar OR Mauritius OR Comoros OR Tanzania OR Seychelles OR Kenya OR Somalia) and Filters applied: from 2020/1/1–2024/1/30.

### Article selection and eligibility criteria

The following types of papers were taken into account for this systematic review and meta-analysis: full text articles, abstracts, and thesis or dissertations that were written only in English, all observational studies that report the prevalence of HBV among pregnant women, outcome ascertainment using rapid test and ELISA methods of diagnostics, the publication year between January 01/2020 to January 30/2024 were included.

Duplicate studies, research done in languages other than English, review articles, and studies conducted before 01/2020 and after 30/2024 were not included in this analysis.

### Outcome of the study

The primary outcome of the study was to determine the pooled prevalence of HBV infection among pregnant women in East Africa, and the secondary outcome of the study was to identify the determinant factors associated with HBV infection among pregnant women in East Africa.

### Data extraction

The data were extracted using a Microsoft Excel spreadsheet by three authors independently (TKT, NMD and GGA). The extracted data were: author names, year of publication, study’s conducted area, study design, sampling technique, outcome ascertainment, total number of participants (samples), number of HBV infected pregnant women, prevalence, and variables related to the prevalence of HBV were also extracted.

### Quality appraisal

Each study quality was discussed and assessed by two independent reviewers (MCA and GAY), and if any deviations arose between these two, the third reviewer (BGY) intervened and a consensus was reached. The studies were assessed using the Newcastle Ottawa Scale (NOS) quality assessment scale [42] and the criteria were representativeness of the sample, sample size, non-response rate, outcome ascertainment, and comparability of the study. The quality assessment was determined by providing a numerical score, with scores 8–9 considered excellent quality, 6–7 very good quality, 4–5 good quality, and below 4 considered poor quality or unsatisfactory, and hence numerical value below 4 excluded from the study (S1 Table in S1 File).

### Statistical analysis and synthesis

The random-effect (DerSimonian) model was used to determine the pooled prevalence and associated factors of HBV infection among pregnant women in East Africa. For those different studies, heterogeneity was assessed by I^2^ statistic, and furthermore, sub-group analysis, and sensitivity analysis were used to analyses potential sources of heterogeneity. The value of I^2^ statistic was an indication of variation across studies, and values of 25%, 50% and 75% were indications of low, medium and high presence of heterogeneity [43]. We assessed the publication bias using the Egger test, and p-values less than 0.05 were an indication of a significant presence of publication bias [44]. As the asymmetry was detected using the funnel plot and Egger’s test; the trim-and-fill method was used to re-estimate the pooled effect size by removing the outlying effect sizes, and then added back into the funnel plot and mirrored on the opposite side to identify the best estimate of the unbiased pooled effect size [45]. The analysis was done using statistical software, STATA version 17.

## Results

### Searching results and included studies

**Fig 1:**
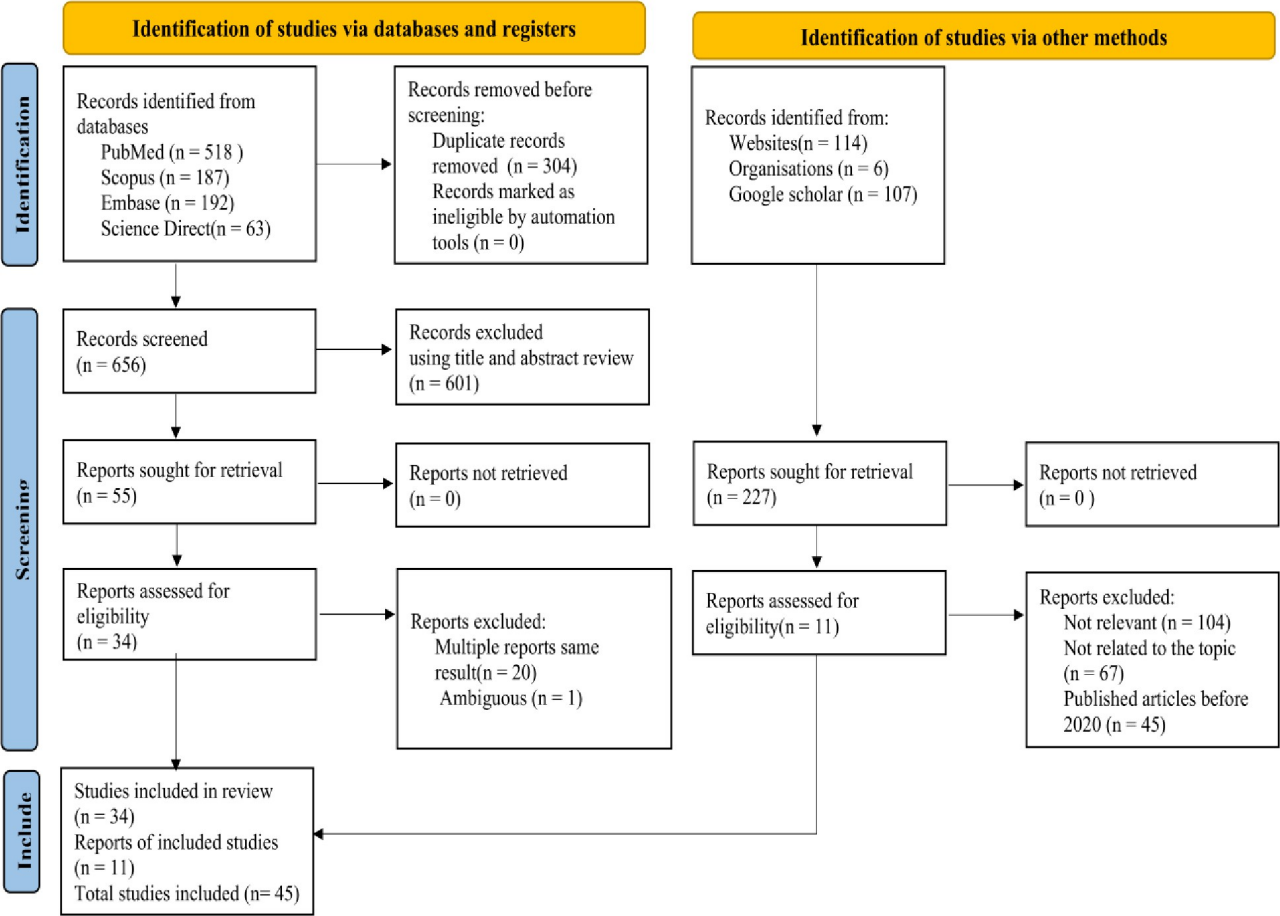
PRISMA 2020 flow diagram for new systematic reviews which included searches of databases, registers and other sources for HBV infection among pregnant women in East Africa [46].

This systematic review and meta-analysis was conducted on the prevalence of HBV and its determinants among pregnant women in East Africa. All studies about the prevalence of HBV among pregnant women were included, and different searching strategies were developed for different databases and a total of 1187 recorded studies 960 from databases, and 227 from other sources were identified. From a total of 960 studies searched using databases, 304 articles were removed before screening because of duplicating, 601 articles removed using titles and abstracts reviews, 1 article was removed due to ambiguity and 20 articles were multiple reports of the same result. From a total of 227 studies searched via other methods, 104 studies were not relevant, 67 studies were not related to the topic, and 45 studies were published before 2020. Finally, 45 studies with a total of 35639 pregnant women (around 792 average pregnant women) were included for systematic and meta-analysis (Fig *1*).

### Characteristics included studies

The studies were conducted in East African countries between 2020 and 2024. 91.11% of the included articles were institutional based cross-sectional studies. The minimum number of pregnant women participated in a study was 124 [12] and the maximum number was 12138 [25]. (Table 1).

**Table 1:** Summary statistics for number of pregnant women.

Characteristics	Number of studies	Minimum	Maximum	Average	Total
Sample size	45	124	12138	791.9778	35639

With regard to publication year, from a total of 45 studies, 9 [10, 18, 19, 24, 28–30, 38, 47] were in 2020, 12 [9, 11, 13, 16, 22, 26, 27, 36, 48–51] in 2021, 14 [12, 14, 15, 20, 21, 23, 31, 32, 37, 39, 40, 52–54] in 2022, and 10 [1, 2, 17, 25, 33–35, 41, 55, 56] in 2023. More than two-third, thirty one (68.89%) of studies were conducted in Ethiopia, three (6.67%) in Uganda, three (6.67%) in Somalia, two (4.44%) in South Sudan, two (4.44%) in Tanzania, one in Djibouti, one in Zimbabwe, one in Mozambique, and one in Kenya (Table 2).

**Table 2:** Characteristics of included studies.

Subgroup Analysis		Number of studies	Percent(%)
Publication Year	2020	9	20.00
	2021	12	26.67
	2022	14	31.11
	2023	10	22.22
	**Total**	**45**	**100**
Country	Djibouti	1	2.22
	Ethiopia	31	68.89
	Kenya	1	2.22
	Mozambique	1	2.22
	Somalia	3	6.67
	South Sudan	2	4.44
	Tanzania	2	4.44
	Uganda	3	6.67
	Zimbabwe	1	2.22
	**Total**	**45**	**100**

### Pooled prevalence of HBV among pregnant women in East Africa

**Fig 2:**
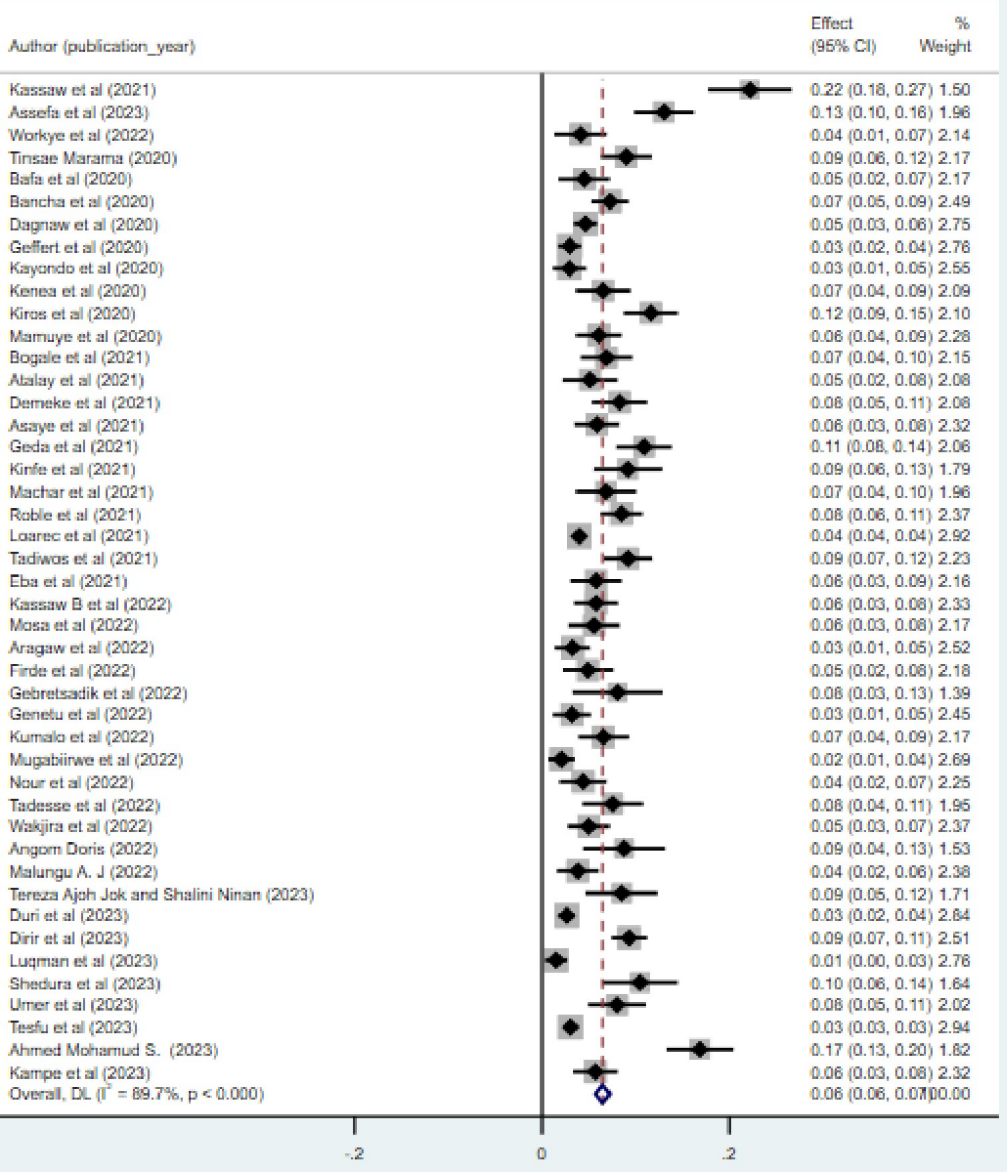
Forest plot for pooled prevalence of HBV infection among pregnant women in East Africa.

As Fig 2 showed from a total of 45 studies, the overall pooled prevalence of HBV among pregnant women in East Africa was 6.0% (95% CI: 6% - 7%) with observed heterogeneity (I^2^ = 89.7%; p-value < 0.0001). The prevalence ranged from the lowest 1.5% reported from Somalia [56] to the highest prevalence of 22.2% reported from Ethiopia [9]. (Fig 2)

### Source of heterogeneity and handling

#### Sub-group analysis

Subgroup analysis was performed using the year of publication, sampling technique, and study design. (Table 3) Nine studies were published in the year 2020, twelve in 2021, fourteen in 2022, and ten in 2023, with the highest prevalence of 8% ((95% CI: 6%, 10%) I^2^ = 91.08%) seen in 2021 and the lowest prevalence 5% ((95% CI: 4%, 6%) I^2^ = 52.52%) observed in 2022. Here, the highest heterogeneity was observed in studies conducted in 2023 (I^2^ = 94.81%). (S1 Fig in S1 File)

**Table 3:** Subgroup analysis for year of publication, sampling technique and study design.

Subgroup Analysis		Number of studies	Pooled Prevalence (%)(95% CI)	P-value	I^2^(%)
Publication year	2020	9	6(4, 8)	<0.0001	84.50%
	2021	12	8(6, 10)	<0.0001	91.08%
	2022	14	5(4, 6)	<0.01	52.52%
	2023	10	7(5, 9)	<0.0001	94.81%
Sampling Technique	Probability Sampling	31	7(6, 8)	<0.0001	84.87%
	Non-Probability Sampling	14	6(4, 7)	<0.0001	90.33%
Study Design	Cross Sectional	41	7(6, 8)	<0.0001	86.64%
	Other	4	4(3, 6)	<0.0001	94.04%

Regarding study design, the highest prevalence 7% ((95% CI: 6%, 8%) I^2^ = 86.64%) was observed under cross-sectional study, and 4% ((95% CI: 3%, 6%) I^2^ = 94.04%) were under other studies. Based on study design highest heterogeneity (I^2^ = 94.04%) was observed studies conducted using other study designs (retrospective, prospective or cohort). (S2 Fig in S1 File)

On the other hand, 31 (68.89%) and 14 (31.11%) of the studies were conducted using probability sampling technique and non-probability sampling techniques respectively. Here the highest prevalence 7% (95% CI: 6%, 8%), I^2^ = 84.87%) was observed studies that were conducted using probability sampling techniques and 6% (95% CI: 4%, 7%), I^2^ = 90.33%) of prevalence was for studies conducted using non-probability sampling techniques. As results showed the highest heterogeneity was observed for studies that were conducted using the non-probability sampling technique. (S3 Fig in S1 File)

### Sensitivity analysis

**Fig 3:**
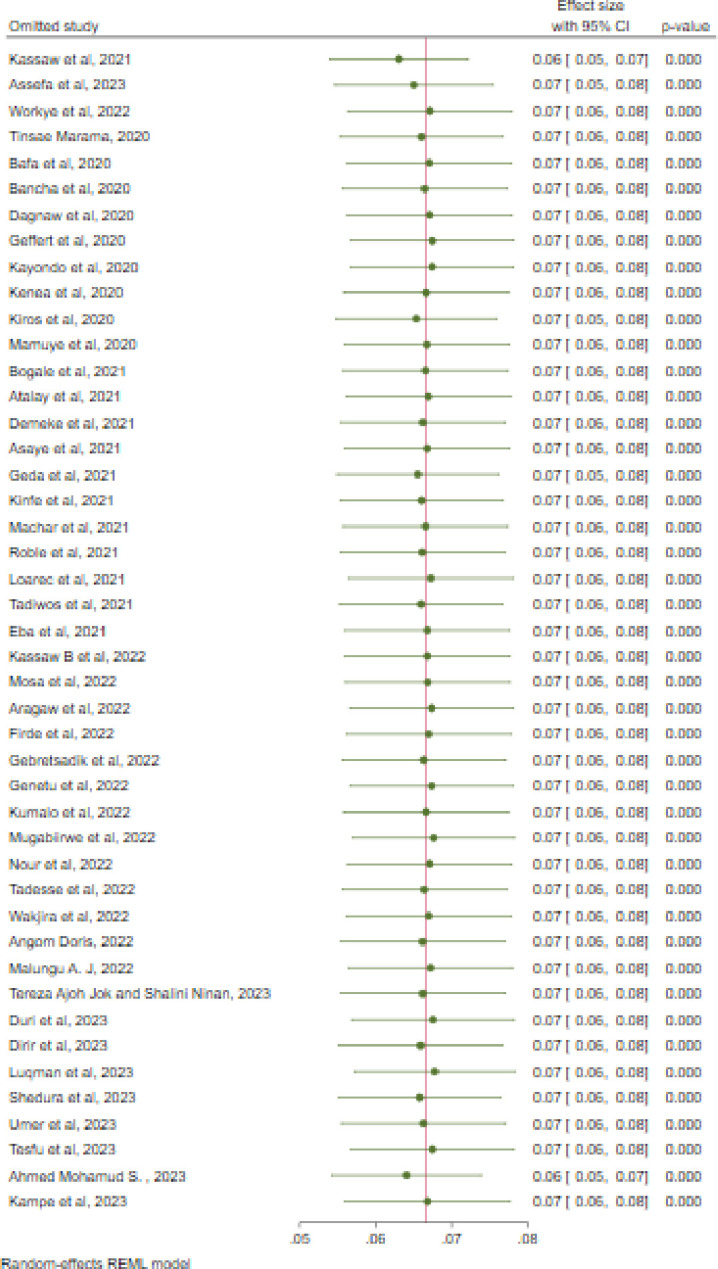
Sensitivity analysis for prevalence of HBV among pregnant women in East Africa.

Sensitivity analysis was conducted, and the result showed that there is no single study whose value lies outside the 95% CI of the overall estimate or pooled prevalence.(Fig 3)

### Publication bias

**Fig 4:**
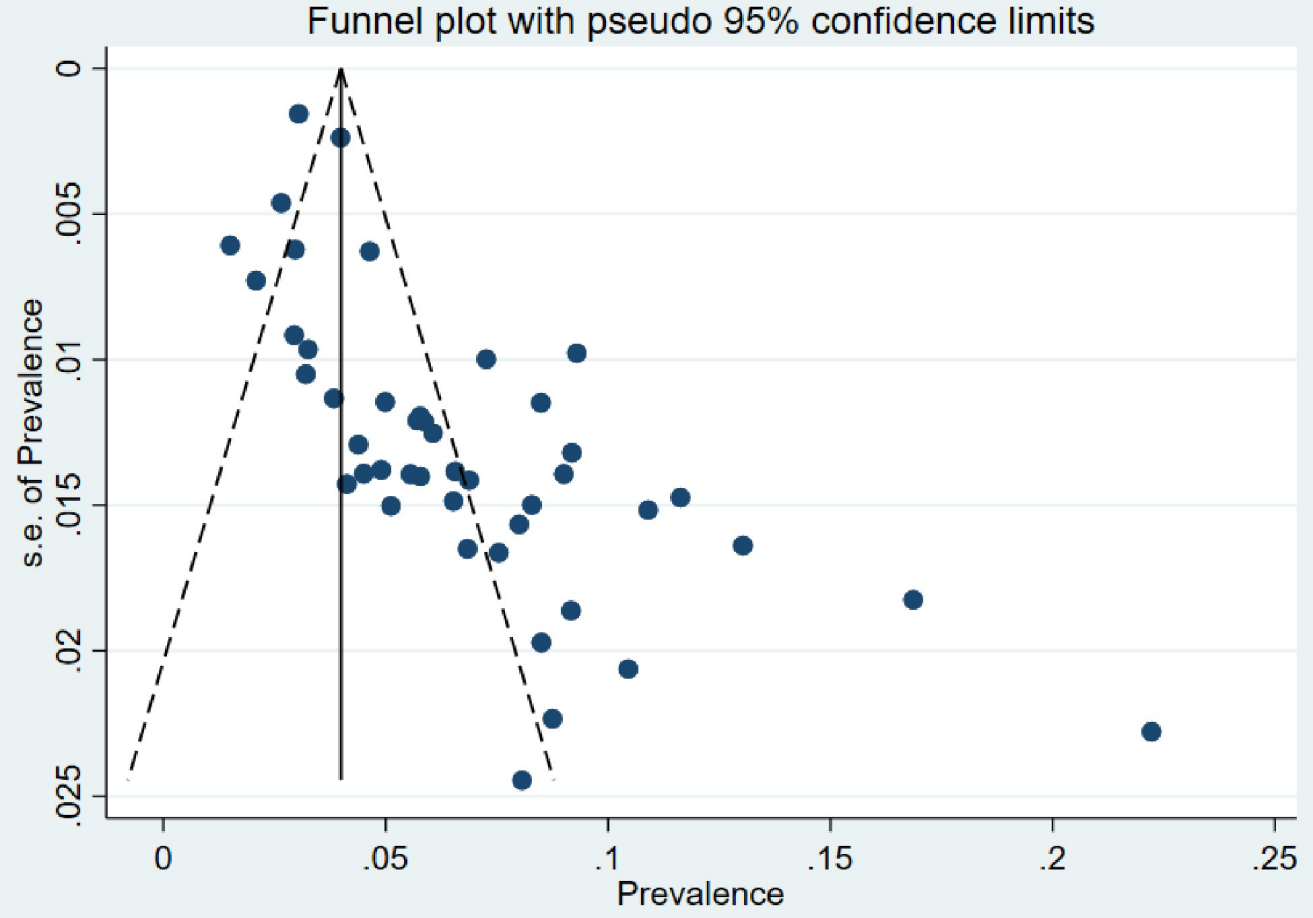
Funnel plot of prevalence with standard error.

The Egger’s test and the funnel plot were conducted. The Egger’s test showed a statistical significant with p-value less than 0.05 and the funnel plot showed an asymmetry of the plot which were an indication of the presence of publication bias. (Fig 4)

This publication bias can be influenced by different factors such as journals may tend to publish studies that show significant results, editors and reviewers may have a bias towards accepting studies with positive results, researchers may manipulate data in order to determine significant findings and many more factors leading to publication bias.

**Fig 5:**
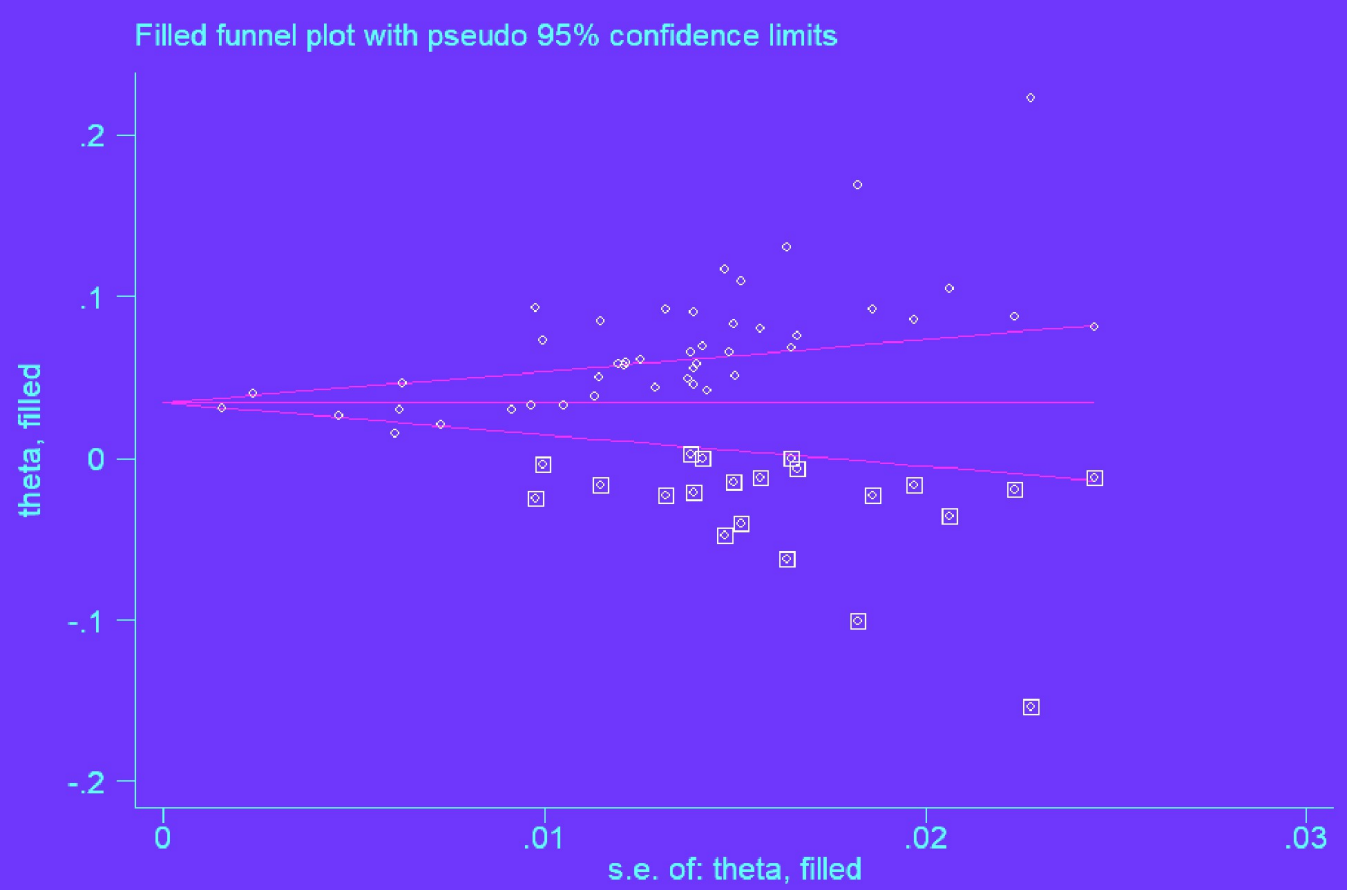
Trim and fill plot.

As the asymmetry was detected using the funnel plot and Egger’s test; the trim-and-fill method (Fig 5) was used to re-estimate the pooled effect size by removing the outlying effect sizes, and then added back into the funnel plot and mirrored on the opposite side to identify the best estimate of the unbiased pooled effect size.

### Pooled determinants of HBV among pregnant women

Here, studies that had two or more common risk factors were taken to identify the associated risk factors with HBV among pregnant women. Table 4 showed the pooled odds ratio of studies that had two and above risk factors in common and surgical procedure, history of having multiple sexual partners, history of body tattooing, history of tooth extraction, abortion history, history of sharing sharp material, blood transfusion, family history of HBV, and history of needle injury were significant risk factors associated with HBV among pregnant women.

**Table 4:** Summary of pooled odds ratio for factors associated with HBV among pregnant women.

Risk Factors	Category	Number of studies	Pooled Odds Ratio(95% CI)	P-value	I^2^(%)
History of surgery	Yes	21	2.14(1.27, 3.61)	0.004	73.2
	No (reference)				
Multiple Sexual partners	Yes	21	3.87(2.52, 5.95)	<0.0001	65.4
	No (reference)				
Genital Mutilation	Yes	9	1.17(0.69, 1.99)	0.554	58.5
	No (reference)				
Body tattooing	Yes	21	2.55(1.62, 4.01)	<0.0001	75.9
	No (reference)				
Tooth Extraction	Yes	18	2.09(1.29, 3.39)	0.003	66.9
	No (reference)				
Abortion History	Yes	25	2.20(1.38, 3.50)	0.001	76.2
	No (reference)				
Sharing Sharp materials	Yes	13	1.88(1.07, 3.31)	0.028	72.8
	No (reference)				
History of Hospital admission	Yes	20	1.60(0.87, 2.92)	0.127	78.5
	No (reference)				
Blood transfusion	Yes	27	2.41(1.62, 3.57)	<0.0001	58.0
	No (reference)				
Family history of HBV	Yes	11	4.87(2.95, 8.05)	<0.0001	53.1
	No (reference)				
Place of residence	Rural	15	1.07(0.77, 1.48)	0.688	23.4
	Urban (reference)				
Marital status	Not Married	8	2.11(0.92, 4.87)	0.079	63.8
	Married (reference)				
Gravidity	One	8	1.07(0.80, 1.44)	0.649	7.6
	Multigravida (reference)				
Parity	One	4			
	Multiparity (reference)				
Sexually transmitted infection	Yes	7	1.34(0.46, 3.89)	0.586	84.8
	No (reference)				
History Needle injury	Yes	5	2.62(1.20, 5.72)	0.015	67.8
	No (reference)				
HIV status	Positive	10	2.46(0.68, 8.87)	0.168	82.1
	Negative (reference)				
Sharing tooth brush	Yes	3	1.11(0.68, 1.83)	0.667	0
	No (reference)				
General risk from Blood Contact	Yes	2	6.39(0.16, 262.98)	0.328	79.1
	No (reference)				
Ear Piercing	Yes	5	1.25(0.71, 2.19)	0.438	41.5
	No (reference)				

A pooled meta-analysis showed that pregnant women who had a history of surgical procedures were 2.14 times more likely to be infected with HBV than who had not (OR = 2.14 (95% CI: 1.27, 3.61), I^2^ = 73.2%). Pregnant women who had multiple sexual partner had a 3.87 higher risk being HBV infection than pregnant women who had no multiple sexual partners (OR = 3.87(95% CI: 2.52, 5.95), I^2^ = 65.4%). Regarding the history of body tattooing, pregnant women who had a history of body tattooing were 2.55 times more likely to be infected than who had not (OR = 2.55 (95% CI: 1.62, 4.01), I^2^ = 75.9%). Similarly, pregnant women who had; history of tooth extraction/dental procedure (OR = 2.09 (95% CI: 1.29, 3.39), I^2^ = 66.9%), abortion history (OR = 2.20 (95% CI: 1.38, 3.50), I^2^ = 76.2%), history of sharing sharp material (OR = 1.88 (95% CI: 1.07, 3.31), I^2^ = 72.8%), blood transfusion (OR = 2.41 (95% CI: 1.62, 3.57), I^2^ = 58.0%), family history of HBV (OR = 4.87 (95% CI: 2.95, 8.05), I^2^ = 53.1%), and history needle injury (OR = 2.62 (95% CI: 1.20, 5.72), I^2^ = 67.8%) had higher risk of HBV infection than their counterparts.

## Discussion

The prevalence of HBV is still high in eastern African countries [5] and HBV in pregnant women can transmit to unborn child during pregnancy [4]. Thus, determining the prevalence of HBV and its determinants among pregnant women improves knowledge, can be considered an input for concerned bodies, to develop prevention strategies and this study was aimed to determine the pooled prevalence and its determinants among pregnant women in East Africa.

Our systematic and meta-analysis used a total of forty five eligible studies with 35639 pregnant women and found that an overall/pooled prevalence of HBV among pregnant women in East Africa was 6.0% (95% CI: 6% - 7%). This finding is close to the study conducted in China, 6.64% [57] and in Nigeria, 6.49% [58]. On the other hand, the pooled prevalence was lower than a study conducted in Ghana, 13.1% [59], in Cammeron, 11.2% [60], and the prevalence was higher than a study conducted in Ethiopia, (4.75%) [7] and (4.7%) [8], in Iran, 1.2% [61]. This difference may be due to differences in participant characteristics, attention given by the government to the virus, study design, cultural and behavioral practices, geographical area, difference in publication year, and difference in applying sampling techniques.

This study identified history of surgical procedure, history of having multiple sexual partners, history of body tattooing, history of tooth extraction, history of sharing sharp materials, history of abortion, blood transfusion, and family history of HBV were among the risk factors of pregnant women for HBV. Pregnant women who had history of surgical procedure had higher risk of acquiring HBV infection than their counterparts. This may be due to weakened immune systems, the use of medical instruments and equipment that are not properly sterilized. This finding is supported by a study [3, 26].

HBV infection transmission increases during sexual activity and the number of sexual partners exposed [1]. Our study showed pregnant women having multiple sexual partners were at higher risk as compared to pregnant women who didn’t have multiple sexual partner. This may be due to the fact that they have had more opportunities to come into contact with the virus through unprotected sex, and the more sexual partners a person has, the higher their risk of exposure to HBV. This finding is in line with studies conducted by [3, 26, 62, 63]. The pregnant women with body tattoo were more likely to be infected with HBV. This is may be due to the possibility for exposure to contaminated needles and unsterilized equipment during the tattooing process. The result is agreed with [7, 13, 64].

Furthermore, the present study found that pregnant women who had a history of tooth extraction were at higher risk of HBV infection. This could be due to bleeding during tooth extraction, which increases the risk of blood exposure, dental instruments not properly sterilized; and dental professionals not following proper infection control procedures. The finding supported by [65]. In addition, pregnant women with an abortion history were more likely to be infected with HBV. The possible reason may be pregnant women who had history of previous abortion may had contact with healthcare facilities that were not properly sterilized, which increases the risk of exposure to communicable diseases. The result agreed with [3, 13, 62].

Pregnant women who had a history of sharing sharp material were at higher risk as compared with those pregnant women who had no history of sharing sharp materials. The possible explanation might be that sharing sharp materials increases the likelihood of exposure to infected blood or body fluids, which can lead to direct contact with infected fluids. This result was consistent with a study conducted in Jigjiga, Ethiopia [26].

The odds of having a history HBV infection among pregnant women who had a history of blood transfusion were higher than those of pregnant women who didn’t have history of blood transfusion. The possible explanation may be the possible explanations may be HBV remains a major risk of transfusion-transmitted infection due to the pre-seroconversion window period, infection with immunovariant viruses, and with occult carriage of HBV infection[66]. This is consistent with a study conducted in Kenya [67], Ethiopia [68, 69]. In addition, pregnant women who had a family history of HBV infection were more likely to be at risk as compared to pregnant women who had no family history of HBV infection. The possible explanation for this may be that pregnant women may have been exposed to the virus at a younger age through close contact with infected family members and genetic factors may play a role in susceptibility to HBV infection. This finding is consistent with a study conducted in Jigjiga, Ethiopia [26].

This study has its own strengths and weaknesses. It is the first study to determine the pooled prevalence of HBV infection among pregnant women in East Africa, which provides a more precise estimate of the overall effect size by integrating data from multiple studies. Among the limitations of this study; firstly, studies written other than in English were ignored, so studies conducted in other languages were missed. Secondly, high heterogeneity was observed. This was an indication that the effect size was different across studies due to study design, geographical region, population, and study year. A random effect model (DerSimonian) and subgroup analysis were conducted to overcome this problem. Thirdly, publication bias was observed due to the researchers of individual studies manipulating data in order to determine significant findings. We used the trim and fill plot procedure to identify the best estimate of the unbiased pooled effect size.

## Conclusion and recommendation

The pooled prevalence of HBV infection among pregnant women in East Africa was an intermediate level and different across countries ranging from 1.5% to 22.2%. History of surgical procedure, history of having multiple sexual partners, history of body tattooing, history of tooth extraction, history of sharing sharp materials, history of abortion, blood transfusion, and family history of HBV were the risk factors responsible for this intermediate level of prevalence among pregnant women in East Africa. The result of this prevalence was an indication of the need for screening, prevention, and control of HBV infection among pregnant women in the region. Therefore, early identification of risk factors, awareness creation on the mode of transmission HBV and implementation of preventive measures are essential in reducing the burden of HBV infection among pregnant women.

## Supporting information

S1 Checklist (DOCX)

S1 File (DOCX)

## Abbreviations

CI: confidence interval
HBV: hepatitis B virus
OR: odds ratio
WHO: world health organizations
ELISA: enzyme-linked immunosorbent assay
