# Supplementary material for: Prevalence of Hepatitis B virus infection and its determinants among pregnant women in East Africa: Systematic review and Meta-analysis
Source: PLoS One. 2024 Jul 12;19(7):e0307102. doi: 10.1371/journal.pone.0307102 (PMC11244773; doi:10.1371/journal.pone.0307102)
Supplement: S1 File — (DOCX) [file pone.0307102.s002.docx]

Table1: Quality assessment for individual studies using Newcastle Ottawa quality assessment scale

|  |  | Selection | | | | Comparability | | Outcome | | Total  score (9) |
| --- | --- | --- | --- | --- | --- | --- | --- | --- | --- | --- |
|  | Year of publication | Representativeness of the sample | Sample size | Non-respondents | Ascertainment of outcome | The study controls for the most important factor | The study control for any additional factor | Assessment of the outcome | Statistical test |  |
| Kassaw et al | 2021 | * | - | * | ** | * | - | * | * | 7 |
| Assefa et al | 2023 | * | * | * | ** | * | - | * | * | 8 |
| Workye et al | 2022 | * | - | * | ** | * | - | * | * | 7 |
| Tinsae Marama | 2020 | - | * | * | ** | * | - | * | * | 7 |
| Bafa et al | 2020 | * | - | * | ** | * | - | * | * | 7 |
| Bancha et al | 2020 | * | * | * | ** | * | - | * | * | 8 |
| Dagnaw et al | 2020 | * | * | * | ** | * | - | * | * | 8 |
| Geffert et al | 2020 | - | * | * | ** | * | - | * | * | 7 |
| Kayondo et al | 2020 | * | - | * | ** | * | - | * | * | 7 |
| Kenea et al | 2020 | * | - | * | ** | * | - | * | * | 7 |
| Kiros et al | 2020 | * | * | * | ** | * | - | * | * | 8 |
| Mamuye et al | 2020 | * | - | * | ** | * | - | * | * | 7 |
| Bogale et al | 2021 | - | - | * | ** | * | - | * | * | 6 |
| Atalay et al | 2021 | * | - | * | ** | * | - | * | * | 7 |
| Demeke et al | 2021 | * | - | * | ** | * | - | * | * | 8 |
| Asaye et al | 2021 | * | - | * | ** | * | - | * | * | 7 |
| Geda et al | 2021 | * | * | * | ** | * | - | * | * | 7 |
| Kinfe et al | 2021 | * | - | * | ** | * | - | * | * | 7 |
| Machar et al | 2021 | * | - | * | ** | * | - | * | * | 7 |
| Roble et al | 2021 | * | * | * | ** | * | - | * | * | 8 |
| Loarec et al | 2021 | - | * | * | ** | * | - | * | - | 6 |
| Tadiwos et al | 2021 | * | * | * | ** | * | - | * | * | 8 |
| Eba et al | 2021 | * | - | * | ** | * | - | * | * | 7 |
| Kassaw B et al | 2022 | * | - | * | ** | * | - | * | * | 7 |
| Mosa et al | 2022 | - | - | * | ** | * | - | * | * | 6 |
| Aragaw et al | 2022 | * | - | * | ** | * | - | * | * | 7 |
| Firde et al | 2022 | * | - | * | ** | * | - | * | * | 7 |
| Gebretsadik et al | 2022 | * | - | * | ** | * | - | * | * | 7 |
| Genetu et al | 2022 | * | - | * | ** | * | - | * | * | 7 |
| Kumalo et al | 2022 | * | - | * | ** | * | - | * | * | 7 |
| Mugabiirwe et al | 2022 | * | - | * | ** | * | - | * | * | 7 |
| Nour et al | 2022 | - | - | * | ** | * | - | * | * | 6 |
| Tadesse et al | 2022 | - | - | * | ** | * | - | * | * | 6 |
| Wakjira et al | 2022 | * | - | * | ** | * | - | * | * | 7 |
| Angom Doris | 2022 | * | - | * | ** | * | - | * | * | 7 |
| Malungu A. J | 2022 | * | - | * | ** | * | - | * | * | 7 |
| Tereza Ajoh Jok and Shalini Ninan | 2023 | - | - | * | ** | * | - | * | - | 5 |
| Duri et al | 2023 | * | * | * | ** | * | - | * | * | 8 |
| Dirir et al | 2023 | * | * | * | ** | * | - | * | * | 8 |
| Luqman et al | 2023 | - | * | * | ** | * | - | * | * | 7 |
| Shedura et al | 2023 | - | - | * | ** | * | - | * | * | 6 |
| Umer et al | 2023 | * | - | * | ** | * | - | * | * | 7 |
| Tesfu et al | 2023 | * | - | * | ** | * | - | * | * | 7 |
| Ahmed Mohamud | 2023 | * | * | * | ** | * | - | * | * | 8 |
| Kampe et al | 2023 | * | - | * | ** | * | - | * | * | 7 |

**Note: one * stands for 1 score**

**Source:** https://cdnlinks.lww.com/permalink/ejgh/a/ejgh_31_9_2019_07_18_nguyen_15743_sdc1.pdf


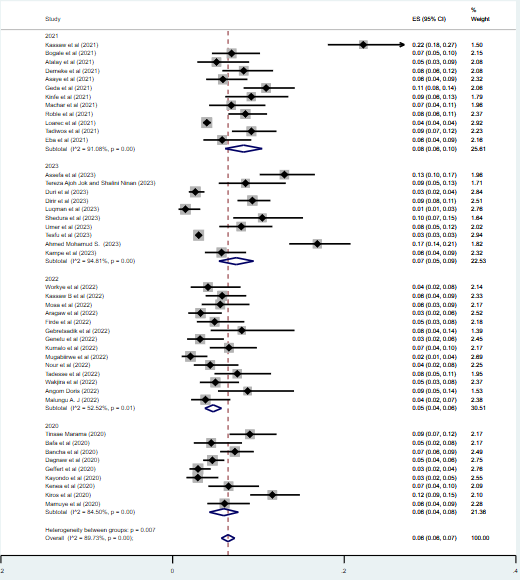


Figure 1: subgroup analysis by year of publication


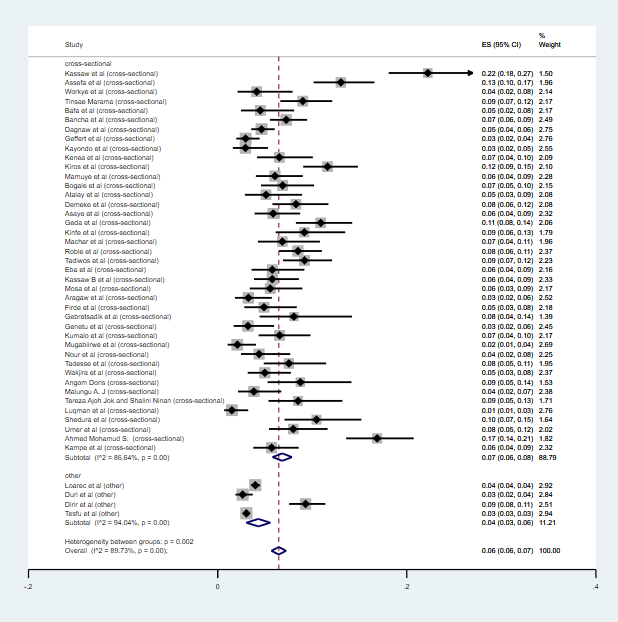


Figure 2: subgroup analysis by study design


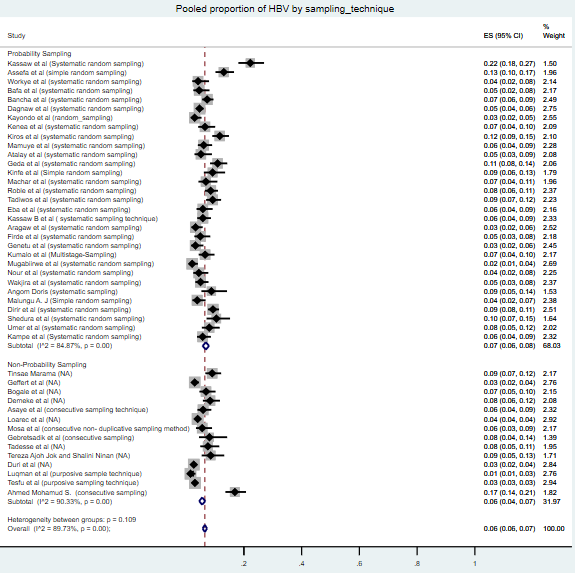


Figure 3: subgroup analysis by sampling technique
